# Cyan Fluorescent Carbon Quantum Dots with Amino Derivatives for the Visual Detection of Copper (II) Cations in Sea Water

**DOI:** 10.3390/nano13061004

**Published:** 2023-03-10

**Authors:** Anastasia Yakusheva, Mohamed Aly-Eldeen, Alexander Gusev, Olga Zakharova, Denis Kuznetsov

**Affiliations:** 1 Department of Functional Nanosystems and High-Temperature Materials, National University of Science and Technology MISIS, Leninsky Prospect 4, 119049 Moscow, Russia; 2 Marine Chemistry Laboratory, National Institute of Oceanography & Fisheries, Kayet-Bey, Al-Anfoushi, Alexandria 5321430, Egypt; 3 Research Institute for Environmental Science and Biotechnology, Derzhavin Tambov State University, 33, Internatsionalnaya Str., 392000 Tambov, Russia

**Keywords:** carbon quantum dots, fluorescence quenching, amino derivatives, water safety, copper sensor, seawater

## Abstract

Amino- and carboxyl-functionalized carbon quantum dots (Amino-CQDs) were synthesized through fast and simple microwave treatment of a citric acid, ethylenediamine and ethylenediaminetetraacetic acid (EDTA) mix. The reproducible and stable optical properties from newly synthesized CQD dispersion with a maximum absorbance spectra at 330 nm and the symmetric emission maximum at 470 nm made the Amino-CQDs a promising fluorescence material for analytical applications. The highly aminated and chelate moieties on the CQDs was appropriate for a copper (Cu^2+^) cation sensor in the linear range from 1 × 10^−4^ mg/mL to 10 mg/mL with a limit of detection at 0.00036 mg/mL by static fluorescence quenching effects. Furthermore, Amino-CQDs demonstrated stable fluorescence parameters for assays in diluted alkali metal solution (Na^+^ and K^+^) and sea water. Finally, a visual sensor, based on Amino-CQDs, was successfully created for the 0.01–100 mg/mL range to produce a colorimetric effect that can be registered by computer vision software (Open CV Python).

## 1. Introduction

Among environmental pollutants, heavy metals are isolated in a separate group of elements due to their toxic effects in ecosystems [1,2,3]. The scientific community have highlighted the most dangerous metals: copper, cadmium, zinc, mercury, lead, chromium, cadmium, arsenic, cobalt and nickel [4,5]. Their dissociated cations affect biological organisms due to their accumulative behavior. Here, we focus on the one of toxic cations—copper. European countries set the safe levels of copper as from 0.00005 to 0.0002 mg/mL for drinking water [6].

Copper cations from water actively transfer to biological organisms and accumulate. The biological accumulation coefficient (BAC) for copper is one of the highest and is equal to six, which means that the concentration of accumulated copper in biological objects is six times greater than in the aquatic medium [7,8,9]. Particular attention should be paid to copper as the main toxic component of antifouling paints for sea and river vessels. Thus, the content of copper cations in open water, marinas and ports is an important indicator, requiring constant measurement [10]. Furthermore, copper pollution exacerbates the effects of acidification and warming for the ocean flora [11]. Consequently, the analytical tools are very promising for copper sensing applications.

Among nanomaterials, the carbon quantum dots are well-studied carbon fluorescence materials, which have been successfully applied to study the contamination of aquatic environments [12]. The sensors are divided into inorganic (heavy metal sensing [13]) and organic (for pesticides [14], herbicides [15], medical drugs [16], proteins [17], vitamins [18]) sensors. Very variable environment with pollutants, e.g., potable water [19], tap water [20], real water sample [21], food products [22] and soil [23], were also tested.

These measuring environments represent the broad applications of carbon quantum dots in analytic chemistry [24]. For example, in one of the papers, Hong-Yi Li et al. presented fluorescent carbon quantum dots for Cr (VI) measurements in wastewater [25]. Another group headed by Debabrata Ghosh Dastidar explored the behavior of CQDs in Tris buffer and chicken plasma with Fe^3+^, Pb^2+^, Cu^2+^, Ca^2+^ and Hg^2+^ ions to prove selectivity for Zn^2+^ in chicken blood plasma [26]. A group of scientists from India used carbon quantum dots for cystine sensing in human blood plasma based on the photoinduced electron transfer fluorescence mechanism [27]. Common to all these papers, the different measuring environments were introduced after testing CQD behavior in model solutions.

Further, studies have shown that the fluorescence properties of CQDs and fluorimetry methodology were effectively applied for a carbon source with ethylenediamine and amino acids, ammonium salts and other amino derivatives [28,29]. Amino functional groups support the complexation chemical reaction between dots and pollutants.

Among the different sensing mechanisms, the fluorescence resonance energy transfer RET (FRET) effect between donor CQD and acceptor (contaminant) has been established for the complexation reaction, especially for heavy metal [30,31]. Hence, the ratio variation in carbon–nitrogen bonds affects the fluorescence emission properties via new chemical bonds [32].

In this work, amino- and carboxy-functionalized carbon quantum dots were obtained and studied as sensors for copper cations in water samples. The mechanism of the interaction of copper cations with Amino-CQDs in aqueous media with a high content of potassium and sodium cations was evaluated in detail. The result showed the stable fluorescent properties of the CQDs, which made it possible to detect copper cations in sea water samples. The classical Stern–Volmer equation for assessment of copper sensing depends on the fluorescence intensity of the Amino-CQDs used. Furthermore, we investigate the ability to create a visual fluorescence sensor by artificial vision for color classification and exhibit the results here.

## 2. Materials and Methods

### 2.1. Reagents and Instruments

In the synthesis procedure, the citric acid (99.8%), ethylenediamine (99.0%) and ethylenediaminetetraacetic acid (EDTA) were used. The metal salts NaCl and KCl, and sea water from the Black Sea were used to test the fluorescence sensor. In all the experiments, deionized water was used as the dispersant.

The synthesized Amino-CQDs were characterized through particle size and zeta using a Zetasizer Nano ZS (ZEN3600; Malvern, Worcestershire, UK) and IR—Fourier spectrometer (Thermo NICOLET 380, Waltham, MA, USA) to study the functional groups. The optical properties were investigated using a UV mini-1240 Shimadzu spectrophotometer (Japan), Agilent Technologies Cary Eclipse Fluorescence Spectrophotometer (USA), portable fluorimeter Sentry-200 (Waukesha, WI, USA) and Origin Lab, Python 3.10 software for processing colorimetric effect.

### 2.2. Synthesis of Amino-CQD

CQD synthesis was performed via microwave irradiation processes as reported previously [33]. Here, we introduced changes in the amino source. The ammonium salts have been replaced with ethylenediamine for prevent the doping effect. First, melted citric acid powder (7 g) was mixed with ethylenediamine in 1:9 molar ratio and heated at 500 W microwave irradiation for 6 min 30 s, then the EDTA in a 1:0.2 molar ratio to citric acid was added and heated again for 3 min to achieve the homogeneous functionalization. The purification procedure was repeated two–three times under 14,500 rpm during 1 h in centrifuge and then filtered through a Millex-LG 0.2 µm IC filter cartridge.

### 2.3. Metal Sensing

#### 2.3.1. Fluorimetry for Copper Cations (Cu^2+^)

The fluorescence quenching effect was investigated via adding the contaminated water probe to an Amino-CQD dispersion with 1000 a.e. of fluorescence intensity. The copper (Cu^2+^) cations were added in the range from 1 × 10^−5^ to 1 × 10^3^ mg/mL concentration in deionized water, KCl, NaCl and sea water solutions to try to create the lowest and most high point of measurements. Further, for analytical measurements, 50 µL of the test sample was added to a cuvette with 1 mL of Amino-CQD dispersion, and then measured. The linear calibration curves and correlation coefficient was calculated as the Stern–Volmer regression for the fluorescence quenching effect according to the following Formula (1):

(1) $$\frac{F_{0}}{F_{i}}=1+{\;K}_{\mathrm{SV}}\left[{\mathrm{Cu}}^{2+}\right]$$

Additionally, the limit of detection was set as the minimal reproducible distinguishable changes in fluorescence intensity by copper cations (Cu^2+^).

#### 2.3.2. Visual Detection of Copper (Cu^2+^) Concentration

Herein, we set up a calibration series to determine the brightest and purest Cyan color of the Amino-CQD dispersion by adding 0.016 mg of Amino-CQD to 1 mL deionized water as shown in Figure S1 and then diluted twice.

The best sample corresponded to the concentration of 0.0005 mg/mL (Figure S1 right column). Then, 50 µL of analytical standard (Cu^2+^) for the reference measurements or copper-contaminated solutions of NaCl, KCl or sea water. Probes were continuously added to the cuvette with the CQD dispersion, and a photograph under UV light (Figure S2) was taken (the position of camera, color temperature (without red channel) was fixed). On each Eppendorf’s photo, the middle region was chosen and pixelated up to five-by-five pixels. The color of each pixel was represented in RGB format, and then blue and green components were used for analysis [34]. Finally, a calibration curve with the dependence of the “saturation” of the blue or green color in the sample dependent on the concentration of copper cations was constructed.

## 3. Results

### 3.1. Primary Characterization of Amino-CQDs

**Figure 1:**
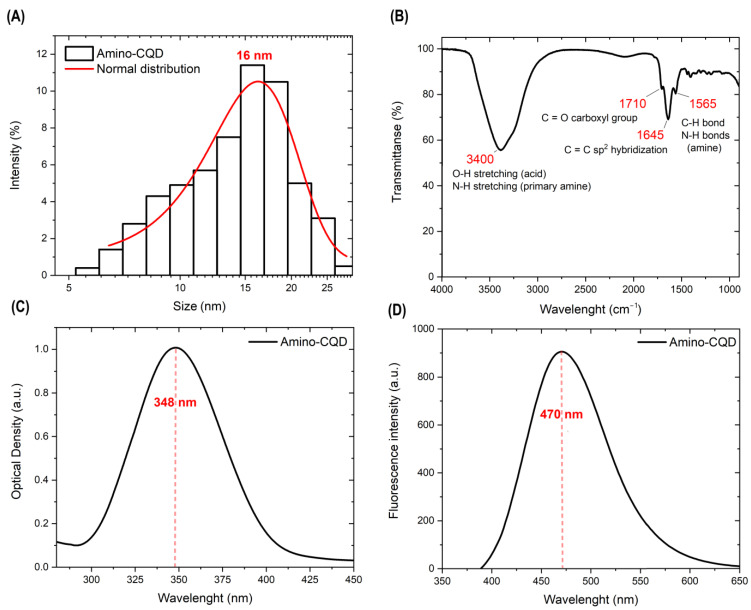
The primary characterization of Amino-CQD dispersion. (**A**) Size distribution, (**B**) IR spectra, (**C**) absorbance spectra and (**D**) emission spectra under 350 ex.

First, we characterized the Amino-CQDs in its storage solution without any changes in concentration and solvent. The dynamic light scattering measurements by Zetasizer Nano ZS (ZEN3600) was used to characterize the average (16 nm) hydrodynamic diameter of the Amino-CQDs and the size distribution from 5 to 25 nm in Figure 1A.

Figure 1B shows the IR spectrum at mid-infrared wavelengths with a peak at 3400 cm^−1^, which is attributed to the -OH wide peak and narrow N-H bond. Additionally, the peak at 1565 cm^−1^ correlated with C-H and N-H bands and confirmed the presence of amino and hydroxy groups in the sample. The C=O from carboxyl groups was also checked as the characteristic adsorption at 1710 cm^−1^ and the carbon core included the atoms in the Sp^2^ hybridization state as a peak at 1645 cm^−1^. The optical properties in Figure 1C exhibit a large excitation peak at 348 nm via an amino functionalization strategy [35]. Here, using ethylenediamine and ethylenediaminetetraacetic acid there is an emission peak at 470 nm in Figure 1D. The reproducibility and stability of the fluorescence properties was confirmed in Figure S3. The zeta potential of the CQDs was 21.8 eV in the 0.0005 mg/mL dispersion.

**Figure 2:**
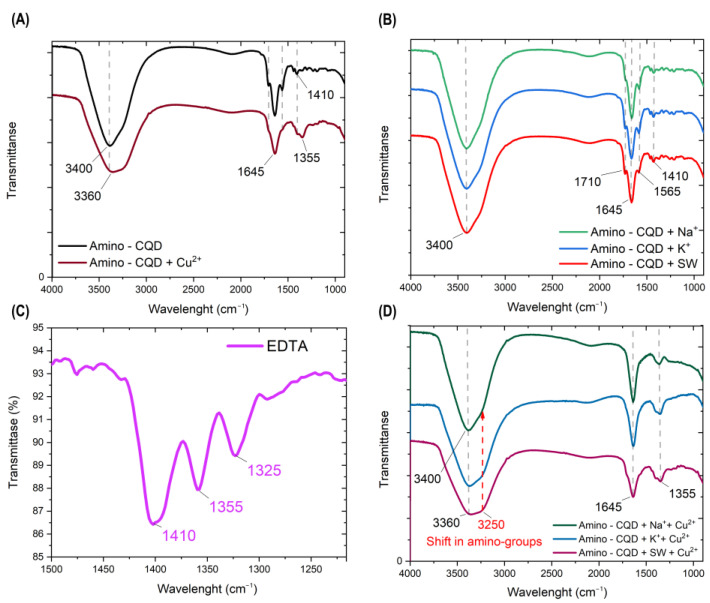
(**A**) The IR spectra of the reaction between Amino-CQD and copper cations, (**B**) Amino-CQD in concentrated alkali ion solution, (**C**) the characteristic line of EDTA in 1250–1500 cm^−1^ region and (**D**) reaction between Amino-CQD and Cu^2+^ in presence of Na^+^ and K^+^ cations.

Next, the IR spectra in Figure 2 show the influence of alkali and copper cations on the functional groups. The peak at 3400 cm^−1^ had a noticeable shift to 3360 cm^−1^ and broader shape with Cu^2+^ coordination near the amino fragment. The shift in amino groups is shown in Figure 2D.

Copper coordination reduced the small peak at 1710 cm^−1^ (C=O) and 1565 cm^−1^ (N-H bond) due to reduction in the response of chemical chain fluctuations.

Therefore, the copper cation deposes the characteristic transmittance from EDTA at 1410 cm^−1^, 1335 cm^−1^ and 1325 cm^−1^ (Figure 2C) as the sum from the nitro and chelate coordinate complexes as a broad peak at 1355 cm^−1^ (Figure 2D). The neighborhood of alkali cations (Figure 2B) did not produce bright changes in the original spectrum (Figure 2A). Additionally, the mobility of Amino-CQDs in ionic solution was explained via polarization fluorescence measurements in Table S1. The data confirm the copper coordination on the Amino-CQD’s surface via the extraordinary rise in polarization and, therefore, the reduction in particle mobility in volume.

### 3.2. Visual Sensor for Copper (II) Cations in Sea Water

**Figure 3:**
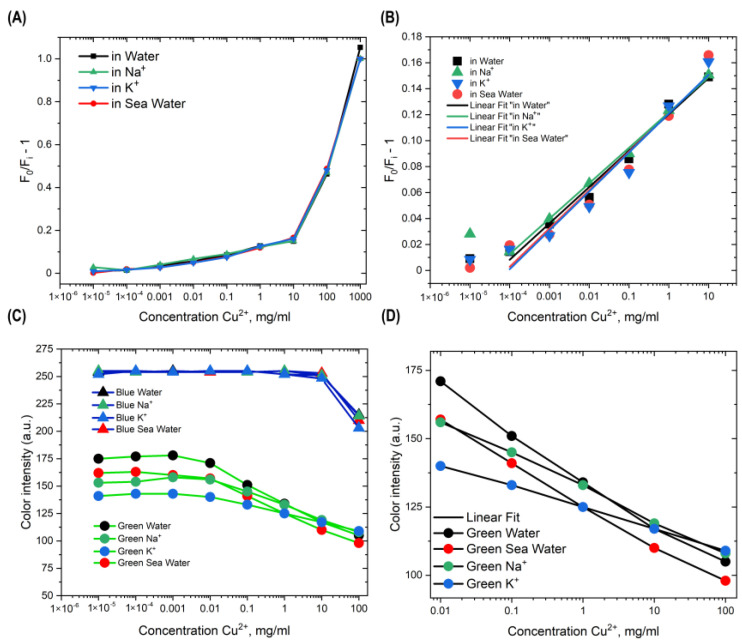
(**A**) Curves reveals the plot between F_0_/F_i_ − 1 and concentration of Cu^2+^ from 1 × 10^−5^ mg/mL to 1000 mg/mL and **(B**) from 1 × 10^−4^ mg/mL to 10 mg/mL as a linear range. (**C**) The calibration curves in green show the reduction effect on Amino-CQD by Cu^2+^ cations from 1 × 10^−5^ mg/mL to 100 mg/mL and (**D**) from 0.01 mg/mL to 100 mg/mL.

The copper sensing was explained as the standard fluorescence quenching effect by copper cations. The calibration curves in Stern–Volmer coordinates are presented in Figure 3A,B.

Table S2 presents the linear correlation with narrow deviations among the cation systems. The intercept and slope are given in the average linear equation in Formula (2):

(2) $$y=0.121+0.1285\left[{\mathrm{Cu}}^{2+}\right]$$

Additionally, the limit of detection via minimal distinguishable changes in fluorescence intensity by copper cations was 0.000036 mg/mL (the min standard was 0.00005 mg/mL).

### 3.3. Automatic Colorimetry Assay via RGB Color Control

Then, for the graphs in Figure 3C,D, we took the middle area of the Eppendorf in the photo of fluorescent dispersion shown in Figure 3S and pixelized the area to 5 × 5 pixels to obtain 25 color data point from the green and blue channels in RGB format (red, green, blue). Amino-CQDs showed a maximum brightness of 255 points for blue (like the cyan color of the reference dispersion), and color reduction when copper cations were added to the sample. In the original procedure, fluorescence quenching was recorded as a change in the green channel. Processing of the data for linear fitting (Table S2) and visual assessment of the sample was able to detect contaminant concentrations from 0.01 mg/mL to 100 mg/mL and from 1 mg/mL to 100 mg/mL, respectively. Testing of the program code are presented in Figure S3.

## 4. Conclusions

In our work, the results demonstrated that the Amino-CQDs with amino- and carboxyl-functionalization could be obtained via microwave synthesis and successfully applied for copper cation sensing in water samples. Therefore, the classical analytical approaches for these measurements showed the potential for copper sensing in alkali cation solutions and sea water. Laboratory measurements showed deviations in optical properties and sensing ability for various ionic media.

The results explain the sensing mechanism for the copper cations based on the fluorescence quenching effect in the linear range from 1 × 10^−4^ mg/mL to 10 mg/mL. The colorimetry assay, assessed by the changes in the green color channel from bright cyan fluorescence Amino-CQD under UV illumination, showed a linear range from 0.01 mg/mL to 100 mg/mL in sensing copper cations, correlating with the admissible copper concentration in natural water.

## 5. Patents

Russian Federation No. 2021137272 “Method for the synthesis of fluorescent carbon nanoparticles for ecological use” (16 December 2021).

## Supplementary Materials

The following supporting information can be downloaded at: https://www.mdpi.com/article/10.3390/nano13061004/s1, Figure S1: The calibration range of Amino-CQD dispersion under UV irradiation for the visual sensing; Figure S2: The visualization of Amino-CQD quenching effect by Cu2+ cations under UV light; Figure S3: The reproducibility and stability of fluorescence properties; Table S1: The polarization fluorescence analysis of Amino-CQD samples in different ionic solutions; Table S2: The linear fitting results for the fluorescence quenching by Cu2+ (A) and the calibration curves for visual test (B).
