# Supplementary material for: Cyan Fluorescent Carbon Quantum Dots with Amino Derivatives for the Visual Detection of Copper (II) Cations in Sea Water
Source: Nanomaterials (Basel). 2023 Mar 10;13(6):1004. doi: 10.3390/nano13061004 (PMC10055749; doi:10.3390/nano13061004)
Supplement: Supplementary file 1 [file nanomaterials-13-01004-s001.zip › nanomaterials-2237070-supplementary.pdf]

nanomaterials MDPI

Supplementary materials

Cyan Fluorescent Carbon Quantum Dots with Amino Derivatives for the Visual Detection of Copper (II) Cations in Sea Water

Yakusheva Anastasia [1,*], Aly-Eldeen Mohamed [2], Gusev Alexander [1,3], Zakharova Olga [1,3] and Denis Kuznetsov [1]

[1] Department of Functional Nanosystems and High-Temperature Materials, National University of Science and Technology MISIS, Leninsky Prospect 4, 119049 Moscow, Russia; nanosecurity@mail.ru (G.A.); olgazakharova1@mail.ru (Z.O.); dk@misis.ru (D.K.)
[2] Marine Chemistry Laboratory, National Institute of Oceanography & Fisheries, Kayet-Bey, Al-Anfoushi, 5321430 Alexandria, Egypt; ~~ma.aly-eldeen@niof.sci.eg~~ m_niof@yahoo.com
[3] Research Institute for Environmental Science and Biotechnology, Derzhavin Tambov State University, 33, Internatsionalnaya str., 392000 Tambov, Russia; nanosecurity@mail.ru (G.A.); olgazakharova1@mail.ru (Z.O.)
* Correspondence: yakusheva.as@misis.ru; Tel.: +7-909-669-3081

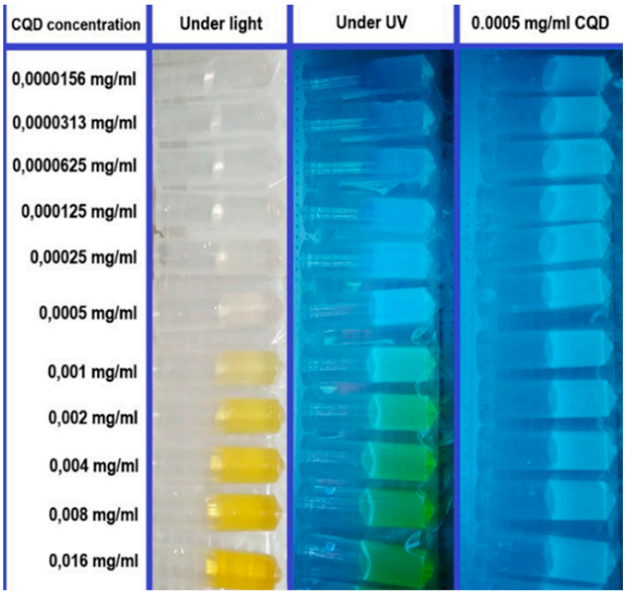

Figure S1. The calibration range of Amino-CQD dispersion under UV irradiation for the visual sensing.

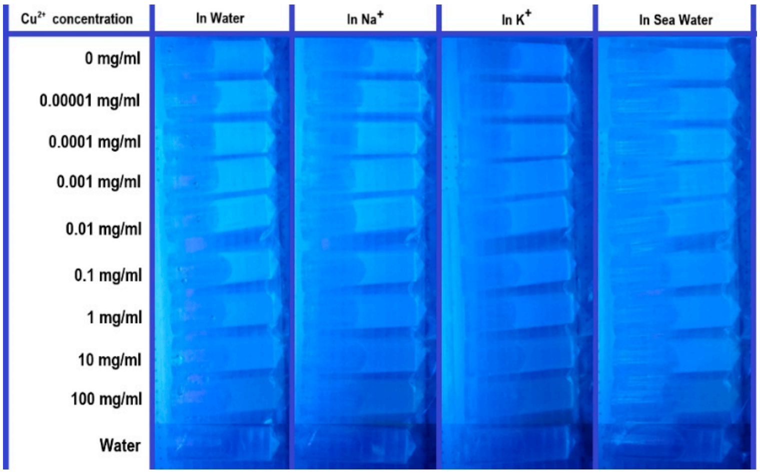

Figure S2. The visualization of Amino-CQD quenching effect by Cu^{2+} cation under UV light.

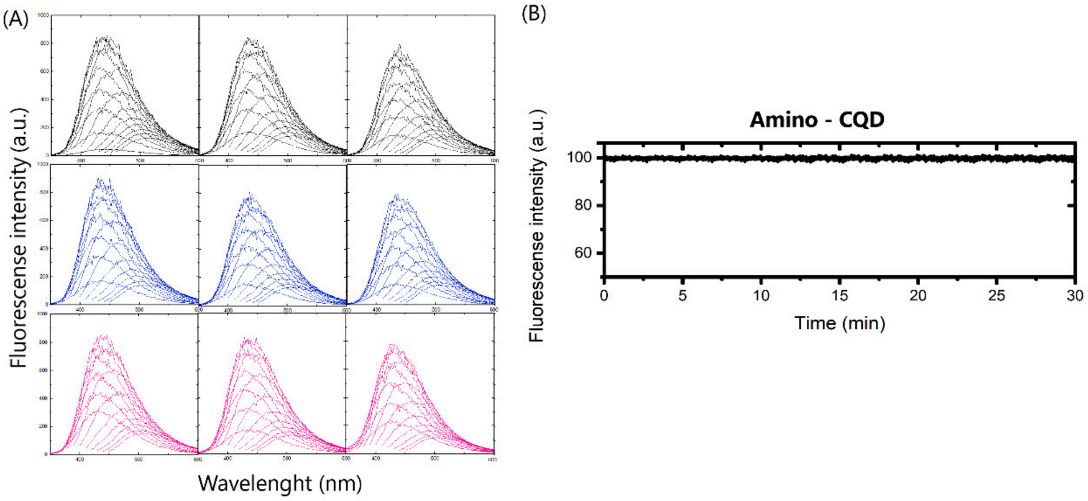

Figure S3 The reproducibility and stability of fluorescence properties.

Table S1. The polarization fluorescence analysis of Amino - CQD sample in different ionic solution.

Sample	Polarization of Fluorescence (a.u.)
Amino-CQD	42.6 ± 0.2
Amino-CQD + Cu^{2+}	181.1 ± 1.8
Amino – CQD in NaCl	46.9 ± 0.3
Amino – CQD in KCl	47.55 ± 0.2
Amino – CQD in Sea Water	49.8 ± 0.3
Amino – CQD in NaCl + Cu^{2+}	183.6 ± 1.8
Amino – CQD in KCl + Cu^{2+}	187.9 ± 1.8
Amino – CQD in Sea Water + Cu^{2+}	193.4 ± 1.8

Table S2. The lineal fitting results for the fluorescence quenching by Cu^{2+} and the calibration curves for visual test.

Stern -Volmer coefficient for the quenching effect in Amino-CQD				
	**In Water**	**In Na+**	**In K+**	**In Sea Water**
Intercept	0,120 ± 0,003	0,121 ± 0,007	0,121 ± 0,001	0,120 ± 0,007
Slope	0,028 ± 0,001	0,029 ± 0,003	0,028 ± 0,001	0,029 ± 0,003
R^2	0,992	0,993	0,998	0,996
**The coefficient for the calibration curves by Green color reduction in Amino-CQD**				
	**In Water**	**In Na+**	**In K+**	**In Sea Water**
Intercept	134,178 ± 4,432	130,464 ± 3,875	123,964 ± 2,200	124,607 ± 3,658
Slope	-11,214 ± 1,618	-6,857 ± 1,415	-4,940 ± 0,803	-10,928 ± 1,335
R^2	0,988	0,970	0,977	0,986
